# Depressive Symptoms and Metabolic Dysregulation Control: A Closer Look at Control Challenges in T2DM Patients

**DOI:** 10.1155/2024/7115559

**Published:** 2024-09-27

**Authors:** Yang Yang, Zhenhua Xing

**Affiliations:** ^1^Department of Cardiovascular Medicine, The Second Xiangya Hospital, Central South University, Changsha, Hunan, China; ^2^Department of Emergency Medicine, Second Xiangya Hospital, Central South University, Changsha 410011, China; ^3^Trauma Center, Second Xiangya Hospital, Central South University, Changsha 410011, Hunan, China; ^4^Emergency Medicine and Difficult Diseases Institute, Second Xiangya Hospital, Central South University, Changsha 410011, China

**Keywords:** depressive symptoms, metabolic dysregulation, PHQ-9, type 2 diabetes mellitus (T2DM)

## Abstract

**Background:** Patients with type 2 diabetes mellitus (T2DM) face an increased risk of developing depression and metabolic dysregulation, which can lead to a higher risk of cardiovascular disease (CVD). However, the relationship between the severity of depression and metabolic dysregulation in patients with T2DM remains unclear. This study aimed to investigate this association using data from the ACCORD-health-related quality of life study.

**Methods and Results:** Patient Health Questionnaire-9 (PHQ-9) scores and medication regimens were assessed at baseline, 1, 3, and 4 years, and HbA1c, blood pressure, and lipid levels were monitored every 4 months over a 4-year period. The severity of depressive symptoms was categorized as none (0–4 points), mild (5–9 points), or moderate–severe (10–24 points) based on PHQ-9 scores. Among the participants, 62% developed depressive symptoms at some point during the 4-year follow-up period, with 21% experiencing persistent depressive symptoms. Participants with moderate-to-severe depression exhibited 0.18% (0.12, 0.24) higher levels of HbA1c, 1.11 mmHg (95% CI, 0.04, 2.15) of SBP, 0.90 mmHg (95% CI, 0.22,1.58) of DBP, and 2.12(95% CI, −0.03, 4.27) mg/dL of LDL, and 0.97 (95% CI, 0.38, 1.56) mg/dL lower levels of HDL compared to their counterparts without depressive symptoms. Moreover, as the severity of depressive symptoms increased, variability in HbA1c and blood pressure levels also increased. Furthermore, patients with more severe depressive symptoms demonstrated suboptimal adherence to medication regimens.

**Conclusion:** Our study found a significant association between depressive symptoms severity and metabolic control in T2DM patients. Greater depressive severity correlated with poorer glycemic, blood pressure, and lipid control, alongside increased variability in these parameters. Additionally, patients with severe depressive symptoms showed suboptimal medication adherence. Addressing mental health in T2DM management is crucial to improve metabolic control and reduce CVD risks.

**Trial Registration:** ClinicalTrials.gov identifier: NCT00000620

## 1. Introduction

Depression is the most common psychiatric disorder worldwide and is prevalent among patients with chronic illnesses [1, 2]. For patients with type 2 diabetes mellitus (T2DM), approximately one in four individuals experience depression severe enough to require clinical intervention, highlighting the critical nature of this issue [3–6]. A meta-analysis has indicated that the rate of depression among diabetic patients is twice as high as in the general population [6]. Additionally, systematic reviews have consistently found that diabetic individuals face a significantly elevated risk of depression compared to nondiabetics [7]. Patients with type 2 diabetes and depression generally face a more severe disease course, greater likelihood of complications, and higher mortality rates compared to those with diabetes alone [3, 8].

The reason behind this association is not completely understood. Both conditions share common etiopathogenic factors, particularly the inflammatory hypothesis. Chronic inflammation has been implicated in the development and progression of both depression and T2DM, suggesting a potential biological link between these conditions [9]. Studies have also found that individuals with depression often exhibit poor medication adherence and engage in unhealthy lifestyles, further complicating their management of T2DM [10–12]. A recent study found that high blood glucose concentrations may be linked to depression. Those with undiagnosed diabetes were nearly twice as likely to have major depression compared to those with normal fasting glucose levels [13]. Furthermore, T2DM frequently suffers from chronic illnesses such as fibromyalgia and rheumatoid arthritis, hypertension, and hyperlipidemia, which are well-established risk factors for depression [14].

Associations between T2DM and depression carry important implications for the clinical care and treatment of both conditions. Although previous studies have found that T2DM patients with depression have worse glucose control, these studies primarily consisted of cross-sectional designs with relatively small sample sizes, resulting in weaker statistical power [12, 15, 16]. Furthermore, fewer studies have investigated the control of lipid levels and blood pressure T2DM, which is often accompanied by metabolic dysregulation. We hypothesized that individuals with T2DM and depression exhibit poorer control over glucose, blood pressure, and lipid levels compared to those without depression. This possible association may partly contribute to the worsened cardiovascular disease (CVD) outcomes observed in patients with T2DM who also suffer from depression; however, this aspect has been relatively underinvestigated. We aimed to explore potential relationships between depressive symptoms and metabolic dysregulation control using a post-hoc analysis of a randomized controlled study.

## 2. Methods

**Figure 1:**
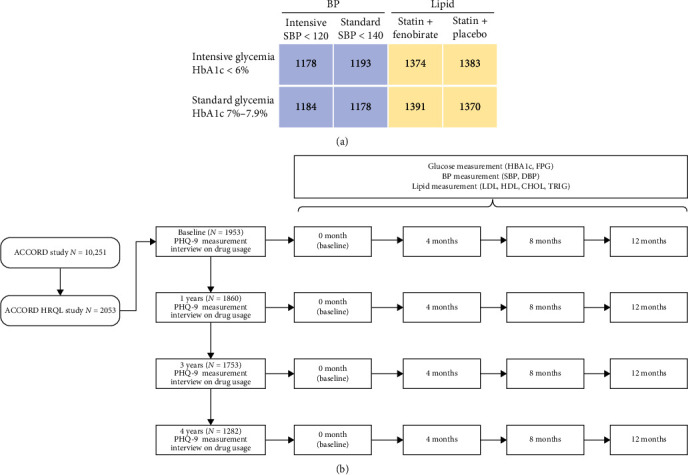
(A) Schematic diagram of the ACCORD study and (B) Schematic diagram of the current study.

The ACCORD study, involving 10,251 participants with a mean age of 62 years and mean HbA1c of 8.3%, aimed to evaluate whether intensified management of blood glucose, blood pressure, and lipid levels could enhance cardiovascular outcomes among individuals with T2DM (Figure 1A). The study was terminated prematurely after an average follow-up of 3.7 years due to the observed increase in cardiac death risk associated with intensive glycemic control. Consequently, all participants shifted to standard glycemic control, with the follow-up continuing as planned [14, 17]. Approval for the ACCORD trial was obtained from an NHLBI review panel and the ethics committees of all participating centers (ethics approval code: HLB01041317a). Notably, participants were not involved in determining research questions, outcome measures, or study design and implementation.

Within the ACCORD study, a subset known as the ACCORD health-related quality of life (HRQL) study aimed to prospectively assess the overall impact of interventions on validated measures of HRQL from participants' perspectives [18]. Specifically, depressive symptoms were evaluated using the nine-item Patient Health Questionnaire-9 (PHQ-9) according to DSM-IV criteria at baseline and at 1, 3, and 4 years during clinical visits within the ACCORD study [8]. The severity of depressive symptoms was categorized as none (0–4 points), mild (5–9 points), or moderate–severe (10–24 points) based on PHQ-9 scores. We divided the participants into three groups: those who never had depressive symptoms during the follow-up period (never), those who had experienced depressive symptoms at some point (ever), and those who had persistent depressive symptoms throughout the follow-up assessments (persistent) Additionally, glucose levels (HbA1c), blood pressure (systolic blood pressure (SBP) and diastolic blood pressure (DBP)), and lipid profiles (LDL and HDL) were assessed every 4 months, with mean levels over the subsequent year reflecting the management of these parameters following PHQ-9 assessments. PHQ-9 scores and the mean levels of HbA1c, blood pressure, and lipids were treated as time-varying variables at baseline and subsequent follow-up time points. Our previous study, consistent with prior research, demonstrated that variability in HbA1c or blood pressure increases the risk of CVD [19–21]. Hence, we also investigated the relationship between PHQ-9 scores and the variability of HbA1c and blood pressure in the following year. HbA1c and blood pressure variability were defined as the intra-individual variability in HbA1c/blood pressure during the subsequent year using average successive variability, which is defined as the average absolute difference between successive values [22]. The association between PHQ-9 scores and subsequent mean levels of these indicators was examined, along with the relationship between PHQ-9 scores and the variability of HbA1c and blood pressure in the following year. Medication usage for glucose, blood pressure, and lipid control was also compared among different depressive symptom severity groups to assess medication adherence.

Baseline participant characteristics, including demographic information, lifestyle factors, and medical history, were collected through questionnaires, interviews, and medical record reviews during recruitment. These characteristics encompassed age, sex, race, duration of T2DM, hypertension, hyperlipidemia, living arrangements, education status, tobacco and alcohol consumption, and medication usage. Smoking status was categorized as never, former, or current, while alcohol consumption was self-reported and quantified in terms of frequency per week. Physiological parameters such as BMI, SBP, DBP, and HR were measured by registered nurses, while lipid profiles, HbA1c, and GFR were assessed by the central laboratory. The design of the current study is depicted in Figure 1B.

Categorical variables were compared using Chi-square analysis, while continuous variables were compared using analysis of variance or Mann–Whitney *U*-tests, depending on their distribution type. Generalized estimating equations with an exchangeable correlation structure were employed to evaluate the relationship between PHQ-9, both as a categorized and continuous variable, and the subsequent mean levels and variability of glucose, blood pressure, and lipids. Two models were utilized: Model 1, adjusted for age, race, and sex, and Model 2, adjusted for age, race, sex, duration of T2DM, education level, history of CVD, heart failure, living arrangement, smoking status, GFR, BMI, and corresponding intervention methods (glucose control for HbA1c, blood pressure control for blood pressure, lipid control for lipid profile). PHQ-9 scores and mean levels of glucose, blood pressure, and lipids were considered as time-varying variables measured at baseline, 1, 3, and 4 years. Sensitivity analyses were also performed by adjusting for medications used for glucose, blood pressure, and lipid control. We also conducted further exploratory research on the relationship between depression and major adverse cardiovascular events (MACE, including cardiac death, MI, and stroke) using Cox regression analysis. All statistical analyses were conducted using Stata/MP Version 17 (StataCorp LP, College Station, TX, USA), and all statistical tests were two-tailed, with a significance level of 5%.

## 3. Results

In the ACCORD study, 2053 participants took part in the ACCORD-HRQL study. Among these, 1953 participants (95%) underwent baseline PHQ-9 measurements. In total, 1860 (91%) completed the 1-year measurement, 1753 (85%) completed the 3-year measurement, and 1282 (62%) completed the 4-year measurement (Figure 1B). Over the 4-year follow-up period, 1267 participants (62%) developed depressive symptoms at some point, with 439 participants (21%) experiencing persistent depressive symptoms. Participants with depressive symptoms tended to be younger, obese, have a history of CVD or heart failure, smoke, exhibit higher levels of HbA1c, LDL, and GFR, and lower levels of HDL compared to their counterparts without depressive symptoms (Table 1).

**Figure 2:**
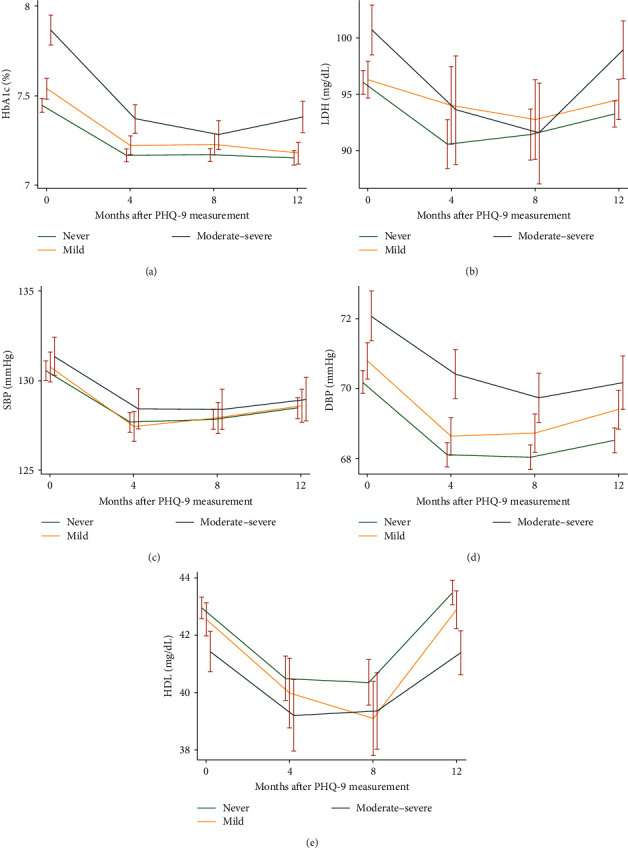
Metabolic profile levels stratified by PHQ-9 category over the subsequent 1 year. (A) HbA1c changes across groups with never, mild, and moderate-to-severe depression; (B) LDL changes across groups with never, mild, and moderate-to-severe depression; (C) SBP changes across groups with never, mild, and moderate-to-severe depression; (D) DBP changes across groups with never, mild, and moderate-to-severe depression; and (E) HDL changes across groups with never, mild, and moderate-to-severe depression.

Participants without depressive symptoms had the lowest mean levels of HbA1c, SBP, DBP, and LDL in the subsequent year, followed by participants with mild depressive symptoms. Participants with moderate-to-severe depressive symptoms had the highest levels of the aforementioned indicators (Figure 2). Similarly, participants without depressive symptoms exhibited the highest levels of HDL, followed by those with mild depressive symptoms and those with moderate to severe depressive symptoms.  Table 2 depicts the association between depressive symptoms and subsequent 1-year mean levels of HbA1c, blood pressure, and lipids after adjusting for confounders. The levels of HbA1c increased with the severity of depressive symptoms (*P* for trend < 0.01). Participants with moderate-to-severe depressive symptoms exhibited 0.18% (95% CI, 0.12, 0.24) higher levels of HbA1c. The PHQ-9 score increased by 1 and was associated with a 0.02% increase in mean levels of HbA1c. Similarly, the levels of SBP, DBP, and LDL increased, and HDL decreased with the severity of depressive symptoms (all *P* for trend < 0.01). Participants with moderate to severe depressive symptoms exhibited 1.11 mmHg (95% CI, 0.04, 2.15) higher levels of SBP, 0.90 mmHg (95% CI, 0.22, 1.58) of DBP, and 2.12 (95% CI,−0.03, 4.27) mg/dL of LDL, and 0.97 (95% CI, −1.56, −0.38) mg/dL lower levels of HDL compared to their counterparts without depressive symptoms. For each increase of 1 in the PHQ-9 score, there was a corresponding increase of 0.09 mmHg in mean levels of SBP, 0.07 mmHg in DBP, and 0.24 mg/dL in LDL, and 0.05 mg/dL lower HDL (all *P*  < 0.01). Sensitivity analyses were also robust by adjusting for medications used for glucose, blood pressure, and lipid control (Table S1).

We also observed that depressive symptoms were associated with higher levels of HbA1c, SBP, and DBP variability (Table 3). With increasing severity of depressive symptoms, larger variability in HbA1c, SBP, and DBP was also observed. We also found that as depressive symptoms severity increased, there was a decrease in the recommended use of antidiabetic medications as per guidelines, including thiazolidinediones (TZD), metformin, sulfonylurea, meglitinide, and insulin (all *P* for trend < 0.05, Table 4). Similarly, as depressive symptoms severity increased, the proportion of thiazide and CCB usage among antihypertensive agents and statin usage among lipid-lowering agents decreased (*P* for trend < 0.05, Table 4). Our exploratory research found depression increased the risk of MACE (Table S2).

## 4. Discussion

This post-hoc analysis of the ACCORD-HRQL study uncovered significant findings regarding T2DM patients at high CVD risk. First, it revealed that T2DM patients experiencing depressive symptoms exhibited poorer management of glucose levels, blood pressure, and lipid profiles. Moreover, as the severity of depressive symptoms increased, these indicators worsened accordingly. Second, the analysis highlighted a correlation between the degree of depressive symptoms and variability in HbA1c, SBP, and DBP, and depressive symptoms were associated with higher risk of MACE. Lastly, it sheds light on the association between the degree of depressive symptoms and suboptimal adherence to medication regimens, particularly concerning antidiabetic medications.

Consistent with previous studies, our research confirms that T2DM patients experiencing depressive symptoms frequently present with hyperglycemia and uncontrolled hypertension [15, 16]. Expanding upon prior findings, our study reveals additional metabolic imbalances in this population, including elevated LDL cholesterol, reduced HDL cholesterol, and heightened variability in HbA1c, SBP, and DBP. These metabolic disturbances are known contributors to increased CVD risk [19–21]. Compared to previous research, we also found a dose–response association between the degree of depressive symptoms and the control of metabolic disturbances. Patients with severe depressive symptoms also exhibited worse metabolic profiles. Our previous study found depressive symptoms changed dynamically in clinical situations [8]. Previous studies primarily consisted of cross-sectional designs with relatively small sample sizes, resulting in weaker statistical power [12, 15, 16]. Our study examined the dynamic changes in depressive symptoms and metabolic profiles using a prospective, repeated-measures design, treating depressive symptoms and metabolic profiles as time-varying variables, thereby ensuring robust statistical power. Moreover, we delve deeper into the investigation by examining medication regimen adherence to elucidate the observed associations. Through this analysis, we offer partial insight into why T2DM patients with depressive symptoms often struggle with blood sugar, pressure, and lipid control. These findings have important clinical implications. Clinical physicians should regularly monitor the psychological status of patients with type 2 diabetes and identify potential depressive symptoms. Timely intervention to promote medication adherence is essential to improve the prognosis of patients with type 2 diabetes and prevent diabetes complications.

The reasons behind the worse metabolic profiles observed in T2DM patients with depression are multifaceted. Depression and T2DM share similar pathophysiological mechanisms, such as inflammation [23]. Recent studies have highlighted the role of inflammation in the development and progression of depression, which is also a key pathophysiological mechanism in metabolic disorders [9]. Patients with depression often experience disruptions in sleep, energy, appetite, and a loss of interest, which undoubtedly impact the management of blood pressure, lipids, and glucose levels, as well as adherence to medication and dietary regimens. Another significant factor is the side effects of antidepressant medications, which can include weight gain and subsequent metabolic abnormalities [3]. Additionally, recent research has found that poor glycemic control further increases the risk of depression. Therefore, the mechanisms linking depression and diabetes are complex and bidirectional [24].

Our present study has limitations. First, it is a post-hoc analysis of an RCT. Consequently, the management of metabolic profiles was strictly controlled by the researchers, which may not fully reflect real-world conditions. In other words, in the real world, T2DM patients with depressive symptoms may exhibit worse metabolic profiles compared to their counterparts in the present study. Second, the present study lacks data on dietary habits and lifestyle modifications. T2DM patients with depressive symptoms may have worse dietary habits and lifestyles, which could influence metabolic profiles [16]. Third, It is important to acknowledge that this study did not involve diagnostic psychiatric interviews to differentiate between depressive symptoms and major depressive disorder among included participants. As a result, the classification of participants was based on self-reported symptoms rather than clinical diagnoses by healthcare professionals. This approach may have implications for the precision and clinical validity of our findings regarding the prevalence and severity of depressive symptoms within the studied population. Fourth, Participants with alcohol consumption who were diagnosed with alcohol use disorder were not included in the present study, which may limit the generalizability of the findings to this population.

## 5. Conclusions

The study revealed that individuals with T2DM and depressive symptoms exhibited poorer control over metabolic profiles, greater variability in HbA1c and blood pressure, and suboptimal adherence to medication regimens. These results emphasize the importance of continuous evaluation and management of both mental health and metabolic profiles in T2DM patients.

## Figures and Tables

**Table 1:** Baseline characteristics of participants based on depression status.

Depressive symptoms status	Never	Ever	Persistent
*N*	686	1267	439
Intensive glucose control	321 (46.79%)	651 (51.38%)	225 (51.25%)
Intensive BP control	175 (52.40%)	321 (50.16%)	108 (49.32%)
Intensive lipid control	180 (51.14%)	308 (49.12%)	108 (49.09%)
Age (years)	63.74 ± 6.29	62.30 ± 6.70*⁣*^*∗*^	61.62 ± 6.58*⁣*^*∗*^
Duration of T2DM (years)	10.42 ± 7.18	11.36 ± 7.96*⁣*^*∗*^	11.32 ± 8.08
Duration of hyperlipidemia (years)	6.21 ± 6.05	5.82 ± 5.49*⁣*^*∗*^	6.02 ± 5.52
Duration of hypertension (years)	9.64 ± 9.23	10.81 ± 9.79	11.02 ± 9.95*⁣*^*∗*^
Sex, male (%)	223 (32.51%)	555 (43.80%)	206 (46.92%)*⁣*^*∗*^
Race, White (%)	426 (62.10%)	822 (64.88%)	295 (67.20%)
CVD history (%)	208 (30.32%)	493 (38.91%)*⁣*^*∗*^	173 (39.41%)*⁣*^*∗*^
Living alone (%)	119 (17.35%)	273 (21.55%)	104 (23.69%)*⁣*^*∗*^
Education (%)	—	*⁣* ^*∗*^	*⁣* ^*∗*^
Less than high school	65 (9.49%)	208 (16.43%)	64 (14.58%)
High-school graduate	185 (27.01%)	324 (25.59%)	114 (25.97%)
Some college	217 (31.68%)	423 (33.41%)	146 (33.26%)
College degree or higher	218 (31.82%)	311 (24.57%)	115 (26.20%)
Heart failure (%)	24 (3.50%)	76 (6.00%)*⁣*^*∗*^	32 (7.29%)
Current Smoker (%)	70 (10.20%)	185 (14.60%)*⁣*^*∗*^	72 (16.40%)*⁣*^*∗*^
Alcohol consumption per week	1.16 ± 2.93	0.80 ± 2.40*⁣*^*∗*^	0.74 ± 2.25*⁣*^*∗*^
BMI (kg/m^2^)	31.37 ± 5.02	33.00 ± 5.50*⁣*^*∗*^	33.97 ± 5.53*⁣*^*∗*^
FPG (mg/dL)	170.41 ± 52.46	179.90 ± 59.03*⁣*^*∗*^	182.39 ± 60.15*⁣*^*∗*^
HbA1c (%)	8.16 ± 1.00	8.35 ± 1.06*⁣*^*∗*^	8.42 ± 1.09*⁣*^*∗*^
Blood pressure			
SBP (mmHg)	136.52 ± 17.34	136.18 ± 16.89	135.53 ± 16.45
DBP (mmHg)	73.82 ± 10.40	74.76 ± 11.16*⁣*^*∗*^	74.65 ± 10.89
Lipid			
LDL (mg/dL)	103.30 ± 32.35	104.63 ± 34.49*⁣*^*∗*^	105.72 ± 35.57
HDL (mg/dL)	42.53 ± 11.38	41.92 ± 11.71*⁣*^*∗*^	41.11 ± 11.25*⁣*^*∗*^
GFR (mL/min/1.73 m^2^)	90.93 ± 37.35	92.16 ± 27.22*⁣*^*∗*^	91.01 ± 26.50*⁣*^*∗*^

Abbreviations: BMI, body mass index; CHOL, total cholesterol; CVD, cardiovascular disease; DBP, diastolic blood pressure; FPG, fasting plasma glucose; GFR, glomerular filtration rate; HbA1c, glycosylated hemoglobin A1c; HDL, high-density lipoprotein; LDL, low-density lipoprotein; SBP, systolic blood pressure; T2DM, type 2 diabetes mellitus; TRIG, triglyceride.

*⁣* ^*∗*^ *p* < 0.05.

**Table 2:** Association between depressive symptoms and subsequent 1-year mean levels of metabolic profile levels after adjusting for confounders.

Metabolic profile	Depression groups	Model 1	Model 2
HbA1c	None	Ref	Ref
	Mide	0.08 (0.03, 0.12)	0.07 (0.02, 0.11)
	Moderate–severe	0.20 (0.14, 0.26)	0.18 (0.12, 0.24)
	*P* for trend	<0.01	<0.01
	PHQ9 continuous	0.02 (0.01, 0.02)	0.02 (0.01, 0.02)

SBP	None	Ref	Ref
	Mide	0.48 (−0.30, 1.27)	0.49 (−0.30, 1.28)
	Moderate–severe	1.12 (0.08, 2.15)	1.10 (0.04, 2.15)
	*P* for trend	<0.01	<0.01
	PHQ9 continuous	0.09 (0.00, 0.17)	0.09 (0.00, 0.17)

DBP	None	Ref	Ref
	Mide	0.29 (−0.21, 0.79)	0.34 (−0.17, 0.84)
	Moderate–severe	0.87 (0.19, 1.55)	0.90 (0.22, 1.58)
	*P* for trend	<0.01	<0.01
	PHQ9 continuous	0.07 (0.02, 0.13)	0.07 (0.02, 0.13)

LDL	None	Ref	Ref
	Mide	−0.01 (−1.55, 1.54)	0.32 (−1.24, 1.88)
	Moderate–severe	1.73 (−0.40, 3.86)	2.12 (−0.03, 4.27)
	*P* for trend	<0.01	<0.01
	PHQ9 continuous	0.19 (0.02, 0.36)	0.24 (0.06, 0.41)

HDL	None	Ref	Ref
	Mide	−0.38 (−0.80, 0.04)	−0.32 (−0.75, 0.10)
	Moderate–severe	−1.03 (−1.62, −0.44)	−0.97 (−1.56, −0.38)
	*P* for trend	<0.01	<0.01
	PHQ9 continuous	−0.06 (−0.11, −0.01)	−0.05 (−0.10, −0.01)

Abbreviations: CHOL, total cholesterol; CVD, cardiovascular disease; DBP, diastolic blood pressure; FPG, fasting plasma glucose; HbA1c, glycosylated hemoglobin, A1c; HDL, high-density lipoprotein; LDL, low-density lipoprotein; PHQ9, nine-item Patient Health Questionnaire; SBP, systolic blood pressure; TRIG, triglyceride.

**Table 3:** Association between depressive symptoms and blood glucose and pressure variability(subsequent 1 year) after adjusting for confounders.

Metabolic profile variability	Depression groups	Model 1	Model 2
HbA1c variability	None	Ref	Ref
	Mide	0.02 (−0.00, 0.04)	0.02 (−0.00, 0.04)
	Moderate–severe	0.10 (0.07, 0.12)	0.09 (0.07, 0.12)
	*P* for trend	<0.01	<0.01
	PHQ9 continuous	0.01 (0.01, 0.01)	0.01 (0.00, 0.01)

SBP variability	None	Ref	Ref
	Mide	0.27 (0.01, 0.54)	0.16 (−0.11, 0.42)
	Moderate–severe	0.72 (0.40, 1.05)	0.56 (0.23, 0.90)
	*P* for trend	<0.01	<0.01
	PHQ9 continuous	0.06 (0.03, 0.08)	0.04 (0.02, 0.07)

DBP variability	None	Ref	Ref
	Mide	0.15 (0.01, 0.29)	0.13 (−0.01, 0.27)
	Moderate–severe	0.53 (0.36, 0.71)	0.48 (0.31, 0.66)
	*P* for trend	<0.01	<0.01
	PHQ9 continuous	0.04 (0.03, 0.05)	0.04 (0.02, 0.05)

Abbreviations: DBP, diastolic blood pressure; FPG, fasting plasma glucose; HbA1c, glycosylated hemoglobin A1c; PHQ9, nine-item Patient Health Questionnaire; SBP, systolic blood pressure.

**Table 4:** Agents for glucose, hypertension, and lipid control based on depressive symptoms status.

**Antidiabetic agents**					
**Depression groups**	**TZD (%)**	**Metformin (%)**	**Sulfonylurea (%)**	**Meglitinide (%)**	**Insulin (%)**

None	42.19	72.82	53.85	12.89	46.21
Mild	40.62	69.37	50.22	10.98	52.02
Moderate–severe	38.42	67.84	46.33%	9.48	54.89
*P* for trend	0.02	<0.01	<0.01	<0.01	<0.01

**Antihypertensive agents**					
**Depression groups**	**Thiazide (%)**	**ACEI/ARB (%)**	**CCB (%)**	***β*-Blocker (%)**	***α*-Blocker (%)**

None	38.96	73.66	23.42	37.57	0.10
Mild	37.10	72.37	23.06	39.55	0.40
Moderate–severe	33.50	72.83	21.41	38.11	2.90
*P* for trend	<0.01	0.41	<0.01	0.43	0.25

**Lipid-lowering agents (LLA)**					
**Depression groups**	**Statin (%)**	**Fibrate (%)**	**Ezetimibe (%)**	**Niacin (%)**	**Other LLA (%)**

None	75.11	4.98	2.87	2.13	0.70
Mild	71.60	4.82	3.58	1.12	1.70
Moderate–severe	69.69	4.96	2.48	2.48	0.01
*P* for trend	<0.01	0.91	0.99	0.73	0.42

Abbreviations: ACEI/ARB, angiotensin-converting enzyme inhibitor/angiotensin receptor antagonist; CCB, calcium channel blocker; LLA, lipid-lowering drugs; TZD, thiazolidinediones.

## Data Availability

The datasets generated and/or analyzed during the current study are available in the BioLINCC repository. URL: https://biolincc.nhlbi.nih.gov.

## Supporting Information

Supporting Information Table S1: Association between depressive symptoms and subsequent 1-year mean levels of metabolic profile levels by further adjusting for medications and Table S2: Association between depression and MACE.
