# Supplementary material for: Depressive Symptoms and Metabolic Dysregulation Control: A Closer Look at Control Challenges in T2DM Patients
Source: Depress Anxiety. 2024 Sep 27;2024:7115559. doi: 10.1155/2024/7115559 (PMC11919147; doi:10.1155/2024/7115559)
Supplement: Supporting Information — Table S1: Association between depressive symptoms and subsequent 1-year mean levels of metabolic profile levels by further adjusting for medications and Table S2: Association between depression and MACE. [file 7115559.f1.docx]

Supplementary Table 1**: Association between depressive symptoms and subsequent 1-year mean levels of metabolic profile levels by further adjusting for medications**

|  |  | **Model2+medications** |
| --- | --- | --- |
| **HbA1c*** | None | Ref |
|  | Mide | 0.09(0.03,0.14) |
|  | Moderate-severe | 0.19(0.13,0.25) |
|  | P for trend | <0.01 |
|  | PHQ9 continuous | 0.02(0.01,0.02) |
| **SBP#** | None | Ref |
|  | Mide | 0.47(-0.29,1.26) |
|  | Moderate-severe | 1.12(0.11,2.13) |
|  | P for trend | <0.01 |
|  | PHQ9 continuous | 0.10(0.01,0.18) |
| **DBP#** | None | Ref |
|  | Mide | 0.33(-0.16,0.82) |
|  | Moderate-severe | 0.89(0.21,1.57) |
|  | P for trend | <0.01 |
|  | PHQ9 continuous | 0.07(0.02,0.13) |
| **LDL&** | None | Ref |
|  | Mide | 0.34(-1.22,1.90) |
|  | Moderate-severe | 2.14(-0.01,4.28) |
|  | P for trend | <0.01 |
|  | PHQ9 continuous | 0.25(0.07,0.43) |
| **HDL&** | None | Ref |
|  | Mide | -0.34(-0.79,0.12) |
|  | Moderate-severe | -1.01(-1.63,-0.38) |
|  | P for trend | <0.01 |
|  | PHQ9 continuous | -0.05(-0.10,-0.01) |

*PHQ9,nine-item Patient Health Questionnaire; CVD,cardiovascular disease; FPG,fasting plasma glucose; HbA1c,glycosylated hemoglobin A1c; SBP,systolic blood pressure; DBP,diastolic blood pressure; CHOL,total cholesterol; TRIG,Triglyceride; LDL,low density lipoprotein; HDL,high density lipoprotein.*

*** further adjusting for TZD,Metformin,Sulfonylurea,Meglitinide,Insulin.

*#* further adjusting for Thiazide, ACEI/ARB,CCB,β-blocker,α-blocker.

*&* further adjusting for Statin,Fibrate,Ezetimibe,Niacin,Other LLA.

Supplementary Table 2, Association between depression and MACE

|  | Model 1 | Model 2 |
| --- | --- | --- |
| None | Ref | Ref |
| Ever | 1.67(1.31,2.16) | 1.50(1.15,1.95) |
| Persistent | 1.95(1.42,2.67) | 1.63(1.16,2.29) |
| PHQ9 continuous | 1.03(1.01,1.05) | 1.02(1.01,1.04) |

MACE,major adverse cardiovascular events.
